# Concomitant Immunity and Worm Senescence May Drive Schistosomiasis Epidemiological Patterns: An Eco-Evolutionary Perspective

**DOI:** 10.3389/fimmu.2020.00160

**Published:** 2020-02-25

**Authors:** Julia C. Buck, Giulio A. De Leo, Susanne H. Sokolow

**Affiliations:** ^1^Department of Biology and Marine Biology, University of North Carolina Wilmington, Wilmington, NC, United States; ^2^Department of Biology, Stanford University, Hopkins Marine Station, Pacific Grove, CA, United States; ^3^Woods Institute for the Environment, Stanford University, Stanford, CA, United States

**Keywords:** schistosoma, aging, senescence, overshoot, praziquantel, concomitant immunity, model, fecundity

## Abstract

In areas where human schistosomiasis is endemic, infection prevalence and egg output are known to rise rapidly through childhood, reach a peak at 8–15 years of age, and decline thereafter. A similar peak (“overshoot”) followed by return to equilibrium infection levels sometimes occurs a year or less after mass drug administration. These patterns are usually assumed to be due to acquired immunity, which is induced by exposure, directed by the host's immune system, and develops slowly over the lifetime of the host. Other explanations that have been advanced previously include differential exposure of hosts, differential mortality of hosts, and progressive pathology. Here we review these explanations and offer a novel (but not mutually exclusive) explanation, namely that adult worms protect the host against larval stages for their own benefit (“concomitant immunity”) and that worm fecundity declines with worm age (“reproductive senescence”). This explanation approaches schistosomiasis from an eco-evolutionary perspective, as concomitant immunity maximizes the fitness of adult worms by reducing intraspecific competition within the host. If correct, our hypothesis could have profound implications for treatment and control of human schistosomiasis. Specifically, if immunity is worm-directed, then treatment of long-standing infections comprised of old senescent worms could enable infection with new, highly fecund worms. Furthermore, our hypothesis suggests revisiting research on therapeutics that mimic the concomitant immunity-modulating activity of adult worms, while minimizing pathological consequences of their eggs. We emphasize the value of an eco-evolutionary perspective on host-parasite interactions.

## Introduction

Parasites are, by their nature, harmful to their hosts. Over the last century, this line of thinking has informed our approach to the treatment and control of human infectious diseases. However, parasites that kill their hosts usually also perish in the process, suggesting that they should instead evolve mechanisms to limit disease burden, and might even protect their hosts under certain circumstances (1). Indeed, recent theoretical and empirical evidence has shown that, to minimize within-host competition, some parasites protect their hosts from infection (2–4). Although these studies examined interactions between parasites of different species, parasites of the same species also compete for host resources, and established individuals might therefore protect their host from invading conspecifics (5, 6). Intraspecific competition might be particularly common among helminths with complex life cycles, because invading individuals are unlikely to be closely related to established adults. The idea that parasites might protect their hosts for their own benefit is not new, but its application to the treatment and control of macroparasitic diseases of humans remains largely unexplored.

Schistosomiasis is a debilitating disease caused by trematodes of the genus *Schistosoma*. Although several schistosome species are known to infect humans, urogenital schistosomiasis is caused primarily by *S. haematobium*, whereas most cases of intestinal schistosomiasis are caused by *S. mansoni* and *S. japonicum*. Together, these parasites infect an estimated 200 million people globally (mostly in sub-Saharan Africa) (7), causing anemia, stunted growth, cognitive impairment, fatigue, infertility, liver fibrosis, and bladder cancer. High global prevalence combined with severe pathological consequences have made schistosomiasis the second most important human parasitic disease worldwide, exceeded only by malaria.

**Figure 1:**
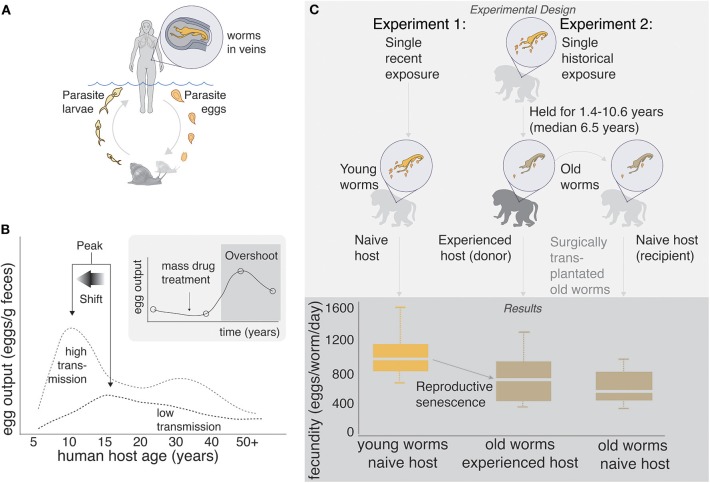
**(A)** Human schistosome life cycle. **(B)** Classic age-intensity curve showing the epidemiological concepts of peak and peak shift. Data are taken from a study in China (8), but similar patterns have been shown in many endemic areas of Africa, Asia, and the Americas. Inset: “Overshoot” is sometimes observed after mass drug administration in areas of high transmission. Data are taken from a study in Senegal (9), but the pattern has been observed in many other areas. **(C)** Experimental evidence of senescence of fecundity in old (1.4–10.6 years) schistosome worms infecting non-human primates. Senescence of fecundity is retained when old worms are surgically transplanted into naïve hosts, suggesting that worms, rather than hosts, are driving the pattern. Data are taken from non-human primate experiments 1: (10) and 2: (11). Most of these trials were conducted on baboons, the putative “best” animal model of human schistosome infection (12).

Schistosomes have complex life cycles. Aquatic snails produce free-living cercariae, which penetrate human skin during bathing and other activities that involve contact with fresh water. Once inside the body, cercariae drop their tails and become schistosomulae, migrating to the lungs and then the liver, and eventually settling in the mesenteric or pelvic vasculature (depending on species) where they pair and mate. A pair of adult worms can produce 300–3,000 eggs per day, about half of which are retained in the host's body. Eggs that are expelled from the body during urination and defecation hatch in the aquatic environment, and free-swimming miracidia infect snails, completing the life cycle (Figure 1A).

The pathology associated with schistosomiasis is caused almost exclusively by the parasite's eggs and the inflammation that they induce. The eggs secrete proteolytic enzymes that trigger a granulomatous immune response (13), thereby facilitating their passage through the endothelial lining of blood vessels and into the lumen of the bladder (*S. haematobium*) or intestines (*S. mansoni* and *S. japonicum*), from which they pass out of the host's body (14). In humans, infection prevalence, worm burden, and egg output generally “peak” around 8–15 years of age and decrease thereafter (15–18), prompting early researchers to speculate about slowly “acquired resistance” (19, 20). Thus, although adults can be infected, children and young adults tend to exhibit the greatest pathology. Furthermore, this peak tends to shift left (i.e., to younger ages) in high transmission areas—the so called “peak shift” (16, 18, 21–24) (Figure 1B).

In 1974, the revolutionary medication praziquantel (PZQ) was introduced to combat schistosomiasis, and global control efforts turned toward drug administration. By killing adult worms, PZQ prevents pathological consequences of the eggs that would otherwise be produced by these worms, and it was hoped that PZQ might eliminate schistosomiasis. Today, mass drug administration (MDA) programs are recommended by the World Health Organization, funded by local governments and private donors, and usually focus on school age children (25, 26). However, although PZQ is reasonably effective at killing adult worms, it fails to prevent reinfection. Therefore, infection prevalence often returns to baseline soon after cessation of treatment, especially in areas of high transmission (27). Although the failure of PZQ to eliminate schistosomiasis is disappointing, it is not especially surprising; for organisms with complex life cycles, population consequences of mortality of a single life history stage may be complicated by density dependence in intervening life history stages (28). Indeed, a recent study suggested that infection risk for humans is controlled by the availability of intermediate snail hosts (29). Furthermore, life-history tradeoffs could allow schistosomes to compensate for PZQ-induced death, either within or between life history stages (30). Most disturbingly, a few recent studies (9, 31, 32) have shown that post-MDA egg counts a year or less after PZQ administration can even exceed pre-MDA egg counts, a phenomenon termed “overshoot” (33, 34).

Peak and peak shift are commonly observed in areas where schistosomiasis is endemic, and overshoot sometimes occurs after the cessation of drug administration (Figure 1B). Here we review several explanations for these patterns that have been suggested previously, and we propose a novel explanation which considers schistosomiasis from an eco-evolutionary perspective.

1) Host-directed acquired immunity

Peak and peak shift are usually assumed to be due to acquired immunity, which is induced by exposure, directed by the host's immune system, and develops slowly over the lifetime of the host (16–18, 18, 19, 23, 34–41), although its mechanism(s) remain controversial. Acquired immunity might depend on exposure to worm antigens that occurs when worms die, rendering adults more resistant than children due to cumulative exposure (16). For instance, death of adult worms, whether natural or due to PZQ-treatment, might elevate levels of antibodies (e.g., IgE and IgG1) whose activity is directed against antigens on the surface membranes of schistosomula (41, 42), or against fecundity of remaining worms (“anti-fecundity immunity”) (43). Alternatively, adult worms might suppress fecundity of conspecifics, termed “density-dependent fecundity” (44). For instance, egg output per worm declines with host age for *S. haematobium* in humans (17, 45), *S. matthei* in sheep (46), and *S. mansoni* in non-human primates (11, 47), but this might not apply to *S. mansoni* in humans (45, 48, 49). It is also possible that children simply have a lower capacity to mount an immune response than do adults, due to “immaturity” of their immune systems or other age-dependent changes in the innate immune response (16, 50, 51). Regardless of the mechanism, acquired immunity is not 100% effective, and even in areas of low transmission, many adults harbor worms (18, 52).

2) Differential exposure of hosts

Several researchers have suggested that the characteristic epidemiological patterns associated with schistosomiasis may have a human behavioral explanation (20, 53). Water contact by children usually exceeds that of adults, both in terms of frequency and duration (54–56). Therefore, children may simply be exposed to more infectious cercariae than adults. However, multiple studies have shown that heavily-exposed adults (i.e., those who are occupationally exposed) are at least partially resistant (38, 39), suggesting that exposure differences cannot fully explain the age-intensity curve. Furthermore, differential exposure cannot explain peak shift or overshoot following the cessation of drug administration.

3) Differential mortality of hosts

Another possible explanation for the characteristic epidemiological patterns associated with schistosomiasis is that they result from the inspection paradox (57). If heavily-infected individuals die at a relatively young age, then they would be undetectable at older ages, causing the appearance that infection intensity declines with age. This possibility results from the nature of most schistosomiasis studies—they are usually conducted as snapshot studies of a whole population, rather than by following individuals over their lifetimes. Although mortality might contribute to peak and peak shift, this explanation cannot fully account for them, as these patterns are detected even in areas where schistosomiasis-induced mortality is rare (58). Furthermore, differential mortality cannot explain overshoot following the cessation of drug administration.

4) Progressive pathology (“tragedy of the commons”)

Another possible explanation for the characteristic epidemiological patterns also arises from the nature of most schistosomiasis studies. Researchers usually quantify egg output in the urine and/or feces, rather than quantifying worm burden or worm fecundity directly. It is possible that fibrosis of the bladder or intestinal wall progresses through time, hindering egg passage from the body. In this case, adults would be expected to excrete fewer eggs than children, even though their worms produce as many eggs as those infecting children. Furthermore, selection pressure should maximize fecundity early in a worm's life, because the chance of an egg reaching the outside environment would deteriorate with time. Although the “tragedy of the commons” might apply to schistosomes (59) and contribute to the characteristic epidemiological patterns, a decrease in egg passage is often accompanied by a decrease in pathology (18), whereas increased pathology would be expected in older individuals if more eggs are retained in the tissues, causing more inflammation and granulomas. Furthermore, autopsy studies [e.g., (48)] have not reported evidence of hindered egg passage, even in individuals with advanced schistosomiasis. Finally, studies that have assessed worm burden via circulating antigens also find the characteristic age-intensity curve (17, 60). Together, these lines of evidence suggest that progressive pathology cannot fully explain observed epidemiological patterns.

5) Concomitant immunity and reproductive senescence

### Concomitant Immunity

To minimize intraspecific competition, adult worms might protect their host against new infections. Termed concomitant immunity, anti-larval immunity, or density-dependent recruitment (not to be confused with density-dependent fecundity), this phenomenon is well-supported in the literature. For example, previously-infected mice and monkeys exhibit resistance to new infections (61–63), and transplant experiments demonstrate that resistance is directed by adult worms (61) for their own benefit (64, 65). Although the mechanism(s) behind concomitant immunity remain controversial [reviewed by Hagan and Wilkins (66)], adult worms produce a rich “secrotome” of potential immunomodulatory molecules (67, 68) that could interact with, or direct, an immune response by the host aimed at larval worms. Adult worms are coated with host antigens, and exhibit multiple mechanisms to evade this immune response, whereas newly invading worms have not yet acquired this protective coat and are more susceptible (65). Thus, long-lived adult worms might protect their host against newcomers, which could benefit adult worms by reducing intraspecific competition and could benefit the host by limiting worm burden.

### Reproductive Senescence

Beyond a certain age, many iteroparous organisms exhibit decreasing fecundity with increasing age (69). Reproductive senescence is well-known in free-living planarians, basal to the trematodes (70), and has also been observed in various schistosome species (46, 47, 71–73), which can live for upwards of 30 years, although 5–10 years is considered an average life expectancy (74). For example, the fecundity of *S. mansoni* declines with increasing host age in non-human primates (47), and transplant experiments suggest that senescence, rather than host-driven immunity, is responsible for this decline (10, 11) (Figure 1C).

When considered from an eco-evolutionary perspective, concomitant immunity and reproductive senescence are logical. To an established adult schistosome, the host represents a vital resource that is worth defending. Incoming larval worms pose a threat, because if they successfully establish, they could reduce the fitness of established adult worms through the “tragedy of the commons,” discussed above (65). However, even if adult worms successfully block larval establishment, they must still compete with other established worms for unhindered passage of eggs into the environment. Therefore, it may be particularly adaptive for schistosomes to maximize their fecundity early in life, but doing so might come at the cost of fecundity later in life, a classic life-history tradeoff (75). Furthermore, due to the complex life cycle of schistosomes, incoming worms are unlikely to be closely related to established worms (76), which should promote self-serving adaptations such as concomitant immunity and senescence (6).

**Figure 2:**
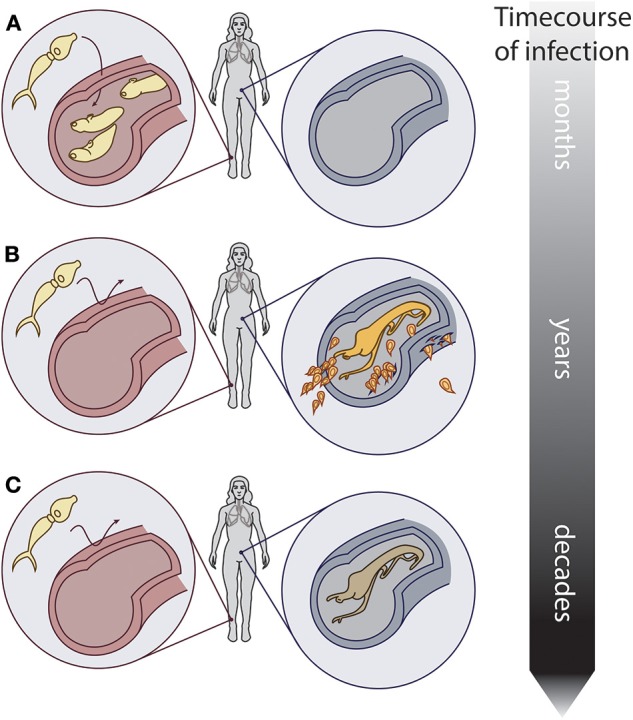
Conceptual hypothesis showing the relationship between worm reproductive senescence, concomitant immunity, and host infection. Over the course of infection in a host: **(A)** Schistosomes infect a naïve host. **(B)** Established adult worms prevent new infections via concomitant immunity, and produce eggs that induce pathology. **(C)** Older worms still prevent new infections via concomitant immunity, but produce few to no eggs due to reproductive senescence. Artwork: Kate Lamy.

Together, concomitant immunity and reproductive senescence could produce epidemiological patterns of peak, peak shift, and overshoot in humans. In endemic areas, young children acquire schistosome infections when they wade into parasite-infested freshwater environments (Figure 2A). As they age, the rate at which new worms are acquired might decrease due to concomitant immunity, but at the same time, established worms mature, and begin producing eggs, which cause pathology (Figure 2B). Finally, when the host reaches maturity, a stable age distribution could be reached by the worm population (i.e., an individual's worm burden is comprised of many old and few young worms), and egg output could slow or even become undetectable due to reproductive senescence (Figure 2C). This series of events could produce the characteristic peak egg output in adolescence, as well as a shift of the peak to younger ages when and where the force of infection is high. Furthermore, if PZQ administration kills reproductively senescent worms and they are subsequently replaced by young, highly fecund worms, this could lead to transiently higher egg output after treatment than before treatment a year or less after chemotherapy treatment—the overshoot phenomenon. A very simple model tracking a cohort of children and their worms as they age confirms that these patterns can be reproduced, given the assumptions of concomitant immunity and reproductive senescence (see Supplementary Material).

## Discussion

Although it is unethical to conduct the kinds of experiments on humans that have provided the best evidence in animals, epidemiological patterns in human populations are largely consistent with concomitant immunity and reproductive senescence (Table S2). For instance, circulating anodic antigen (CAA), a schistosome-specific, gut-derived glycoprotein, is secreted by adult worms and can be used as a relative measure of worm burden. For both *S. mansoni* and *S. haematobium*, worm burden (as measured by CAA) often declines after a peak during adolescence (17, 60). Although concomitant immunity alone would produce a plateau (Supplementary Material), worm burden might decline due to differential exposure, differential mortality, and/or partial long-term immunity. Similarly, the fecundity (eggs/worm) of both *S. mansoni* and *S. haematobium* declines with host age, at least until adolescence (17, 48), consistent with reproductive senescence. Further evidence for reproductive senescence comes from a study that compared egg count and CAA in an area of long-standing endemicity and an area experiencing a newly-established epidemic (60). These authors reported lower worm fecundity in the endemic area than the epidemic area, as would be expected if worm fecundity declines with worm age. A recent study failed to find evidence of overshoot in children under age seven (77), which suggests that young children might harbor younger, highly fecund worms than older hosts, providing further evidence for reproductive senescence. Finally, in areas of low prevalence, many humans harbor worms, but shed eggs at levels undetectable by urine filtration or stool assay (52). This recently-reported phenomenon, termed “egg-negative/worm-positive schistosomiasis,” might result from concomitant immunity and reproductive senescence, and could impact schistosomiasis control efforts. Although more research is needed, epidemiological patterns in humans are largely consistent with concomitant immunity and reproductive senescence.

Mechanisms underlying long-recognized epidemiological patterns such as the characteristic age-intensity curve have inspired considerable speculation, but few explanations have reconciled peak, peak shift, and rapid overshoot parsimoniously (see Table S2). Here we present a novel hypothesis that can reconcile these patterns—namely that natural senescence of fecundity, in combination with concomitant immunity, could underlie peak, peak shift, and overshoot, irrespective of the host-directed immune response. To our knowledge, no model has previously incorporated worm parameters that change with worm age. Although we are not the first or only researchers to suggest an active role of worms in producing characteristic epidemiological patterns (3, 59, 61, 65, 78), this viewpoint has apparently lost favor and has in recent decades been supplanted by more host-centric hypotheses. Nevertheless, recent evidence that the host immune response changes with host age and exposure history does not logically disprove earlier findings that live adult worms are key to resistance (61). We do not know which of the hypotheses presented here will ultimately prevail as the key drivers of epidemiological patterns in naturally-infected humans, but we hope that the hypothesis space is broadened, minds are re-opened to the possible active role of the worms in the host-parasite relationship, and confirmation bias does not hinder progress.

## Conclusions and Future Directions

Here we suggest that, in combination, concomitant immunity and reproductive senescence could allow old worms to protect the host against new infections, while minimizing pathological consequences of infection. Future studies should confirm whether living adult worms produce resistance similar to that produced by dead worms or worm antigens, and whether aged worms offer the same protection as young, highly fecund worms. If our hypothesis is correct, then it could have profound implications for the treatment and control of schistosomiasis. Specifically, it suggests that treating existing infections without addressing the potential for rapid reinfection may result in transient overshoot in high-transmission areas, because old worms with low egg output might protect their hosts against new infections, while minimizing egg production, and therefore egg-associated pathology. Furthermore, if living adult worms confer protection via concomitant immunity, then sterile adult worms or therapeutics that mimic the concomitant immunity-modulating activity of adult worms might offer promising avenues to be explored in the ongoing effort to control schistosomiasis. We emphasize that the ideas presented here are plausible, but currently represent nothing more than a hypothesis, which nevertheless is logical when schistosomiasis is considered from an eco-evolutionary perspective. We also note that our hypothesis is not mutually exclusive, and could be acting in concert with any of the other explanations presented here to produce observed patterns. Finally, our hypothesis must be tested experimentally before it is used to inform treatment and control efforts. Despite these caveats, we encourage future efforts to apply eco-evolutionary thinking to the treatment and control of schistosomiasis.

## Data Availability Statement

All datasets generated for this study are included in the article/Supplementary Material.

## Author Contributions

JB drafted the manuscript and edited the supplementary information. GD developed the model contained in the supplementary information, and edited the manuscript. SS developed the idea and edited both the manuscript and Supplementary Material and wrote Table S2.

### Conflict of Interest

The authors declare that the research was conducted in the absence of any commercial or financial relationships that could be construed as a potential conflict of interest.
