# Supplementary material for: Concomitant Immunity and Worm Senescence May Drive Schistosomiasis Epidemiological Patterns: An Eco-Evolutionary Perspective
Source: Front Immunol. 2020 Feb 25;11:160. doi: 10.3389/fimmu.2020.00160 (PMC7053360; doi:10.3389/fimmu.2020.00160)
Supplement: Supplementary file 1 [file Data_Sheet_1.pdf]

Supplementary Material

A simple age-structured model for worm senescence and concomitant immunity

We here define a simple age-structured model to describe how the number of adult worms and their age distribution in an initially naïve cohort of human hosts change through time as a function of the force of infection (FOI; i.e., the rate at which new worms are recruited per unit time), and worm mortality (i.e., the rate at which worms are lost per unit time). To simulate reproductive senescence, we assume that worm fecundity (i.e., the rate of egg production per mated female worm) decreases monotonically with worm age (Figure S1). To simulate concomitant immunity, we assume that recruitment of new worms declines with the number of adult worms harbored by the host at a given time.

We use the model to track the total number of eggs produced per unit time, computed as the sum across all worm ages classes of the number of female worms in that worm age class times the worms' age-specific fecundity. Because simulation time also tracks the age of the human subjects in our model, we can use the model to generate age-intensity curves, and to examine egg output before and after chemotherapy (praziquantel administration).

Model structure

Our model builds upon classic epidemiological models for schistosomiasis infection, specifically on the equation tracking the mean parasite load, w (mean number of worms per human host; see for instance eq.3.1a in (1)) in a cohort of initially naïve children:

$$dw/dt = \beta \cdot C - \mu \cdot w \qquad \text{(eq. S1)}$$

where C is the concentration of cercariae in the water, the transmission rate, β, is an aggregated parameter accounting for the rate of human water contacts per unit time and the probability of infection given human contact with cercariae-contaminated waters, and μ is a constant within-host parasite mortality rate per unit time. Because we aim to build the simplest possible model, ignoring the complexities introduced by specifically tracking snail populations, $R = \beta \cdot C$ approximates the force of infection from cercariae in the water to humans contacting that water, assuming the cercarial pool is constant.

Here we considered a discrete-time version of eq. (1S) which tracks worm age x, namely:

$$\boldsymbol{w}_{t+1} = \mathbf{M} \bullet \boldsymbol{w}_t + \boldsymbol{R}(\boldsymbol{w}_t) \qquad \text{(eq. S2)}$$

where:

- $\boldsymbol{w}_t = [w_{1,t}, w_{2,t}, \ldots, w_{n,t}]^T$ is a vector for age structure, i.e., number of worms (males + females) of age 1, 2,… n^+ (w_{n+} indicating the number of worms of age n or older) at time t (the symbol "T" indicates the transpose operator);

- $\boldsymbol{R(w_t)}$ = $[\beta(\mathbf{w}_t)\cdot C, 0, 0,...,0]^T$ is a vector tracking recruitment of new worms where the aggregated parameter β is not constant as in eq. (S1) but a monotonic declining function of total parasitic load;
- **M** is an "*n* x *n*" square matrix with the first row equal to zero and the sub-diagonal elements equal to σ=exp(-μ), i.e., the fraction of worms surviving from time *t* to time *t+1;*
- the symbol "•" indicates matrix multiplication.

$$\boldsymbol{w}_t = \begin{bmatrix} w_{1,t} \\ w_{2,t} \\ w_{3,t} \\ \dots \\ w_{n-1,t} \\ w_{n^+,t} \end{bmatrix} \qquad \boldsymbol{M} = \begin{bmatrix} 0 & 0 & 0 & \dots & 0 & 0 \\ \sigma_1 & 0 & 0 & \dots & 0 & 0 \\ 0 & \sigma_2 & 0 & \dots & 0 & 0 \\ 0 & 0 & & \dots & 0 & 0 \\ \dots & \dots & \dots & \dots & \dots & \dots \\ 0 & 0 & 0 & \dots & \sigma_{n-1} & \sigma_{n^+} \end{bmatrix} \qquad \boldsymbol{R(w}_t) = \begin{bmatrix} \beta \cdot C \cdot e^{-\delta \cdot \sum_x w_{x,t}} \\ 0 \\ 0 \\ \dots \\ 0 \\ 0 \end{bmatrix}$$

This simple model is thus a variant of a Leslie (i.e., age-dependent) model (2,3) with density-dependent exogenous recruitment.

We track worm abundance through time in a single cohort of human subjects that all begin exposure at the same age (here assumed to be 4 years old), and we track egg output before and after praziquantel administration. We assume that the concentration of cercariae *C* is constant over the entire simulation time, whereas the transmission rate is defined as an exponentially decreasing function of parasitic load (i.e., concomitant immunity regulates worm recruitment rate), namely:

$$\beta(\mathbf{w}_t) = \beta_0 \cdot exp(-\delta \cdot \textstyle\sum_{x=1}^{n} w_{x,t}) \qquad \text{(eq. S3)}$$

where β_0 is the maximum transmission rate, δ is a parameter measuring the strength of concomitant immunity, and the symbol Σ indicates summation over the entire worm age distribution to compute total parasite burden at time *t*.

Egg output was computed as follows:

$$E_t = \varepsilon \cdot \textstyle\sum_{x=1}^{n} f_x \cdot ½\, w_{x,t} \qquad \text{(eq. S4)}$$

where ε is the fraction of eggs that are excreted, f_x is the per capita fecundity, an S-shaped decreasing function of age *x* (Figure S1), and the coefficient ½ is used to derive the number of females in the population assuming an average 1:1 sex ratio.

Model parameterization

Time *t* was measured in months. Simulations not reported here show that using a finer time step - for instance, one week or one day - did not change the outcome of the analysis.

Table S1 – model parameters

**Parameter, *unit***	**Description**
t [*months*]	time [*time step*]
$w_{x,t}$	mean number of adult worms (males + females) of age x at time t per host
1:1	mean female-to-male sex ratio
LE = 5 *years*	life expectancy
$\mu = 1/\text{LE} = 0.2\ y^{-1}$	mortality rate
$\sigma = e^{-\mu/365} = 0.983$	fraction of adult worms surviving to the next month
$f_{max} = 350$ *eggs/day*	maximum per capita worm fecundity
$x_{\text{med}} = 5$ *years*	worm age at which fecundity is half the maximum value
$\eta = 10$ *months*	a parameter inverse to the slope of the age-dependent fecundity function
$\beta_0 C = 1$ *per month*	product of cercariae concentration C and maximum transmission rate β_0
$\delta = 0.05$	strength of density-dependent exponential reduction in recruitment induced by concomitant immunity
$\varepsilon = 0.9$	Fraction of eggs excreted from human host

Mean worm life expectancy (*LE*) was set to 5 years, and mortality rate was computed as $\mu = 1/LE = 0.2$ [y^{-1}] = $1.66 \cdot 10^{-2}$ [$months^{-1}$]. The fraction of live adult worms at time t surviving to the next month was computed as $\sigma = exp(-\mu) = 0.983$ per month. Assuming a lower life expectancy (e.g., 3 years) changed neither the qualitative outcome of the simulations nor the conclusions of this paper.

Per-capita fecundity was assumed to be described by the following S-shaped, monotonically decreasing function of age:

$$f_x = f_{\text{max}}/(1+\exp((x-x_{\text{med}})/\eta)$$

where maximum per-capita fecundity, f_{max}, was set to 350 eggs per day and x_{med} and η (a parameter inverse to the slope of the function) were set to 5 [years] and 10 [months], respectively, to simulate a per-capita fecundity rate fairly constant until age 3 with a smooth drop in egg output between age 3 and 7 (Figure S1).

We focus here on *Schistosoma haematobium*, although a similar model could apply to *S. mansoni* or *S. japonicum.* To simulate egg count per 10 ml of urine, we assume a urine volume equal to 1500 ml per day and a 10% egg retention per day in the human body.

The product of maximum transmission rate and cercariae concentration in the water, $\beta_0 C$, is set to 1 and the parameter measuring the strength of concomitant immunity δ is set to 0.05.

We also ran a sensitivity analysis for values of $\beta_0 C$ equal to 0.01, 0.1 (low FOI), and 2 and 5 (high FOI) times the reference value ($\beta_0 C = 1$) and for stronger concomitant immunity, namely $\delta = 0.25$.

In the simulation, we assume children are first infected at a transmission rate equivalent to that in the rest of the ages in the cohort (for the constant $\beta_0 C$ assumption to be valid) beginning at age 4.

The model was implemented in R version 3.5.1 and the script is available upon request.

Results

The simple model presented here can parsimoniously reproduce epidemiological patterns of peak and peak shift in the age-intensity curve, as well as overshoot after praziquantel administration.

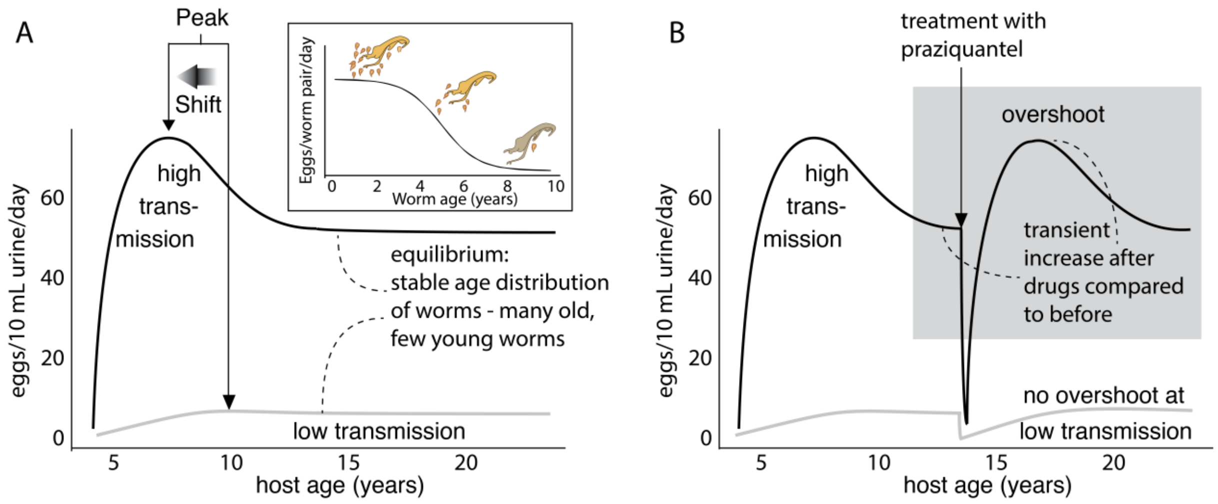

Figure S1. A) The graph tracks egg output in a cohort of hosts across host ages: each host in the cohort has a population of worms of differing ages that contribute to total egg output in that host. Therefore, at equilibrium, a stable age distribution in the worm population within each host, with many old and few young worms, leads to relatively low and unchanging egg output after several years of endemic transmission/infection. Inset shows the assumption governing worm reproductive senescence with increasing worm age (in years). B) The epidemiological pattern of overshoot after drug treatment is predicted in high but not in low transmission settings, consistent with patterns observed in endemic sites whereby some populations, but not all, exhibit overshoot (i.e., overshoot is more likely at ‘persistent hotspots’ of transmission).

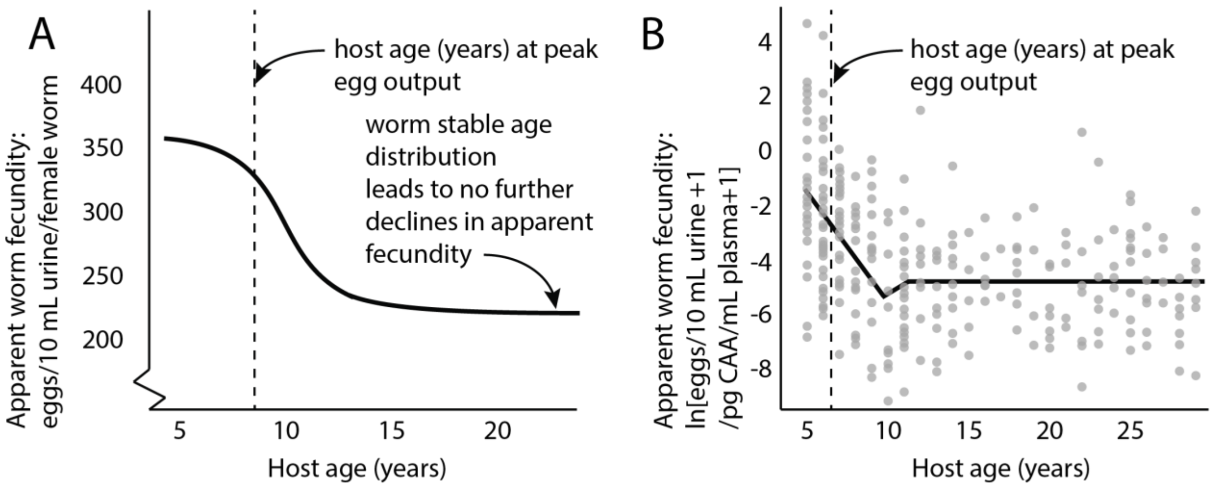

Figure S2. A) Model-predicted average apparent worm fecundity by age of host computed as the overall egg output (i.e., the sum of egg output from all female worms of all ages in each age class of host) divided by the total number of female worms in hosts of that age. B) Field observed change in worm fecundity over age of hosts in a Malian population (reproduced from data in (4)) with worm fecundity proxied as the number of eggs/10mL urine divided by the CAA pg/mL plasma (a gut-associated glycan constitutively released by adult worms) in each host of various ages. Points are results for individual hosts; the line is the result of a piecewise linear regression, with separate regressions for children of age <11 and older individuals of age >=11 (see (4) for details). Vertical dashed line represents the age at maximum egg output (peak) for A) this iteration of our model, or B) field data (4).

Model caveats and limitations

Our model tracks worm burden through time in a single, initially naïve cohort of hosts exposed to cercariae-contaminated waters, but does not describe the full life cycle of schistosome worms. Therefore, it does not track the reduction in cercarial concentration expected to occur following repeated, community-wide drug treatments, nor does it follow variations in egg output with host age-dependent behavior or susceptibility. Accordingly, we assumed cercarial concentration C and exposure rate β_0 were set to constant background levels not affected by praziquantel administration or host factors. We chose this simplification deliberately in order to demonstrate whether peak, peak shift and overshoot could be reproduced, even in the absence of differences in exposure or risk across age classes. Additionally, for simplicity, we ignored any possible Allee effects (the drop in mating probability in small worm populations due to difficulty of finding a mate (5–8)). Future iterations of the model could consider these complexities, among others, to observe the measure of influence of concomitant immunity and reproductive senescence with respect to other potential hypotheses about the mechanisms driving epidemiological patterns.

References

1. Woolhouse M, Taylor P, Matanhire D, Chandiwana S. Acquired immunity and epidemiology of Schistosoma haematobium. *Nature* (1991) **351**:757–759. doi:10.1038/351757a0

2. Leslie PH. Some Further Notes on the Use of Matrices in Population Mathematics. *Biometrika* (1948) **35**:213–245. doi:10.2307/2332342

3. Allen LJS. A density-dependent Leslie matrix model. *Math Biosci* (1989) **95**:179–187. doi:10.1016/0025-5564(89)90031-X

4. Wilson S, Jones FM, van Dam GJ, Corstjens PLAM, Riveau G, Fitzsimmons CM, Sacko M, Vennervald BJ, Dunne DW. Human Schistosoma haematobium antifecundity immunity is dependent on transmission intensity and associated with immunoglobulin G1 to worm-derived antigens. *J Infect Dis* (2014) **210**:2009–2016. doi:10.1093/infdis/jiu374

5. Arakala A, Hoover CM, Marshall JM, Sokolow SH, Leo GAD, Rohr JR, Remais JV, Gambhir M. Estimating the elimination feasibility in the "end game" of control efforts for parasites subjected to regular mass drug administration: Methods and their application to schistosomiasis. *PLoS Negl Trop Dis* (2018) **12**:e0006794. doi:10.1371/journal.pntd.0006794

6. Stephens PA, Sutherland WJ, Freckleton RP. What Is the Allee Effect? *Oikos* (1999) **87**:185–190. doi:10.2307/3547011

7. May RM. Togetherness among Schistosomes: its effects on the dynamics of the infection. *Math Biosci* (1977) **35**:301–343. doi:10.1016/0025-5564(77)90030-X

8. Hoover CM, Sokolow SH, Kemp J, Sanchirico JN, Lund AJ, Jones IJ, Higginson T, Riveau G, Savaya A, Coyle S, et al. Modelled effects of prawn aquaculture on poverty alleviation and schistosomiasis control. *Nat Sustain* (2019) **2**:611–620. doi:10.1038/s41893-019-0301-7
