# Supplementary material for: Concomitant Immunity and Worm Senescence May Drive Schistosomiasis Epidemiological Patterns: An Eco-Evolutionary Perspective
Source: Front Immunol. 2020 Feb 25;11:160. doi: 10.3389/fimmu.2020.00160 (PMC7053360; doi:10.3389/fimmu.2020.00160)
Supplement: Supplementary file 2 [file Table_2.docx]

Supplementary Material

Table S2: Hypotheses to explain classic epidemiological patterns: 1) host-directed acquired immunity, 2) differential exposure of hosts, 3) differential mortality, 4) progressive pathology, 5) concomitant immunity and reproductive senescence. For each observed epidemiological pattern (columns), an X indicates whether a hypothesis can plausibly explain it, and a (?) indicates a potential to explain it under some but not all circumstances. The number of patterns explained is a measure of parsimony. The final two columns list evidence supporting and refuting each hypothesis: modeling studies, human epidemiological studies, *in vitro* studies, animal model studies, and meta-analyses. Among the hypotheses examined, the most parsimonious are those involving immunity (host-directed or concomitant): (1a) host-directed anti-worm immunity, (1b) host-directed anti-fecundity immunity, and (5) concomitant immunity plus reproductive senescence (the hypothesis presented in this paper). However, no single hypothesis is unequivocally supported by the evidence.

| Hypothesis category | Hypothesis description | Host role | Worm role |  |  | Can it explain? | | |  | |  | |  | |  |  | |  | |  | Evidence for | | Evidence against | |  |
| --- | --- | --- | --- | --- | --- | --- | --- | --- | --- | --- | --- | --- | --- | --- | --- | --- | --- | --- | --- | --- | --- | --- | --- | --- | --- |
|  |  |  |  | Peak egg output at school age | Peak shift | Overshoot | Peak worm burden at school age | Decline in worm burden among older adults >30-40yrs | | Decline in (fecal/ urinary) egg output among older adults  >30-40yrs | | Decline in apparent worm fecundity in adults | | Pre-treatment intensity is predictive of post-treatment intensity in individuals | | | Observed lower reinfection in older individuals | | Observed fast rebound in human population after PZQ (“hotspots”) | | |  | |  | |
| (1) host-directed acquired immunity | (a) delayed, host-driven "classical" acquired immunity to dead/dying worms | active | passive | X | X | (?)* | X |  | |  | |  | | X | | | X | |  | | | Modeling:[^1-3^](#_ENREF_1)  Epidemiology:[^3-13^](#_ENREF_3)  Lab animal:  In vitro: [^14^](#_ENREF_14)  Meta-analysis: | | Modeling: [^15^](#_ENREF_15)^,^ [^16^](#_ENREF_16)  Epidemiology:[^17-21^](#_ENREF_17)  Lab animal: [^9^](#_ENREF_9)^,^ [^22^](#_ENREF_22)  In vitro:  Meta-analysis: [^23^](#_ENREF_23) | |
|  | (b) delayed, host-driven, "anti-fecundity" acquired immunity driven by either: epitopes revealed as worms die, OR epitopes on eggs (or both?) | active | passive | X | X | (?)* | X |  | |  | | X | | X | | | X | |  | | | Modeling: [^3^](#_ENREF_3)^,^ [^24^](#_ENREF_24)^,^ [^25^](#_ENREF_25)  Epidemiology: [^7^](#_ENREF_7)^,^ [^8^](#_ENREF_8)^,^ [^26^](#_ENREF_26)^,^ [^27^](#_ENREF_27)  Lab animal:[^28-30^](#_ENREF_28)  In vitro:  Meta-analysis: | | Modeling:  Epidemiology:[^31^](#_ENREF_31)  Lab animal:[^32^](#_ENREF_32)  In vitro:  Meta-analysis: | |
|  | (c) innate susceptibility changes due to puberty hormones in people | passive | passive | X |  |  | X |  | |  | |  | | X | | | X | |  | | | Modeling:  Epidemiology:[^33^](#_ENREF_33)  Lab animal:  In vitro:  Meta-analysis: | | Modeling:  Epidemiology:[^7^](#_ENREF_7)  Lab animal:  In vitro:  Meta-analysis: | |
|  | (d) cross reactivity of invading cercariae to adult worm antigens; depending on the length of time those antibodies circulate, it could be concomitant or acquired. | passive | passive | X | X |  | X |  | |  | |  | | (?) | | | (?) | | (?) | | | Modeling:  Epidemiology: [^34^](#_ENREF_34)  Lab animal:  In vitro:  Meta-analysis: | | Modeling:  Epidemiology:  Lab animal:  In vitro:  Meta-analysis: | |
|  | (e) cross reactivity of invading cercariae to egg antigens decrease recruitment success; depending on the length of time those antibodies circulate, it could be concomitant or acquired. | passive | passive | X | X |  | X |  | |  | |  | | (?) | | | (?) | | (?) | | | Modeling:  Epidemiology:  Lab animal:  In vitro: [^35^](#_ENREF_35)  Meta-analysis: | | Modeling:  Epidemiology:  Lab animal:  In vitro:  Meta-analysis: | |
| (2) differential exposure | differential exposure - water contact patterns | active | passive | X |  |  | X | X | | X | |  | | X | | | X | | X | | | Modeling: [^36^](#_ENREF_36)^,^ [^37^](#_ENREF_37)  Epidemiology: [^38^](#_ENREF_38)  Lab animal:  In vitro:  Meta-analysis: | | Modeling: [^39^](#_ENREF_39)  Epidemiology:[^11^](#_ENREF_11)  Lab animal:  In vitro:  Meta-analysis: | |
| (3) differential mortality of hosts | differential mortality of hosts means that those alive at any one time is biased: older adults are observed if they are less infected because highly infected people already died | passive | passive | X | X |  | X | X | | X | |  | | (?) | | | (?) | | (?) | | | Modeling:  Epidemiology:  Lab animal:  In vitro:  Meta-analysis: | | Modeling:  Epidemiology:  Lab animal:  In vitro:  Meta-analysis: | |
| (4) progressive pathology | progressive pathology that impacts egg passage | passive | passive | X | X |  |  |  | | X | |  | | (?) | | | (?) | | (?) | | | Modeling:  Epidemiology: [^26^](#_ENREF_26)  Lab animal:  In vitro:  Meta-analysis: | | Modeling:  Epidemiology:[^40^](#_ENREF_40)  Lab animal:  In vitro:  Meta-analysis: | |
| (5) concomitant immunity + reproductive senescence of worms – this paper’s hypothesis | host-parasite immune interaction/worm life history whereby live adult worms interact with host immune system to prevent superinfection; worms experience reproductive senescence over time | active | active | X | X | X | X |  | |  | | X | | (?) | | | (?) | | X | | | Modeling: [^41^](#_ENREF_41), [^37^](#_ENREF_37)^,^ [^42^](#_ENREF_42) [^7^](#_ENREF_7), this paper  Epidemiology: [^8^](#_ENREF_8)^,^ [^10^](#_ENREF_10)^,^ [^26^](#_ENREF_26)^,^ [^40^](#_ENREF_40)^,^ [^43^](#_ENREF_43)^,^ [^44^](#_ENREF_44)  Lab animal: [^22^](#_ENREF_22)^,^ [^29^](#_ENREF_29)^,^ [^30^](#_ENREF_30)^,^ [^45-52^](#_ENREF_45)  In vitro:  Meta-analysis: | | Modeling:  Epidemiology: [^5^](#_ENREF_5)^,^ [^11^](#_ENREF_11)^,^ [^31^](#_ENREF_31)^,^ [^53^](#_ENREF_53)^,^ [^54^](#_ENREF_54)  Lab animal:  In vitro:  Meta-analysis: | |

*slower than observed

**References**

1. Anderson RM, May RM. Herd immunity to helminth infection and implications for parasite control. Nature. 1985;315:493-6.

2. Woolhouse ME, Taylor P, Matanhire D, Chandiwana SK. Acquired immunity and epidemiology of Schistosoma haematobium. Nature. 1991;351:757-9.

3. Mitchell KM, Mutapi F, Savill NJ, Woolhouse ME. Explaining observed infection and antibody age-profiles in populations with urogenital schistosomiasis. PLoS Comput Biol. 2011;7:e1002237.

4. Hagan P, Blumenthal UJ, Dunn D, Simpson AJ, Wilkins HA. Human IgE, IgG4 and resistance to reinfection with Schistosoma haematobium. Nature. 1991;349:243-5.

5. Kabatereine NB, Vennervald BJ, Ouma JH, Kemijumbi J, Butterworth AE, Dunne DW, et al. Adult resistance to schistosomiasis mansoni: age-dependence of reinfection remains constant in communities with diverse exposure patterns. Parasitology. 1999;118 ( Pt 1):101-5.

6. Black CL, Mwinzi PN, Muok EM, Abudho B, Fitzsimmons CM, Dunne DW, et al. Influence of exposure history on the immunology and development of resistance to human Schistosomiasis mansoni. PLoS Negl Trop Dis. 2010;4:e637.

7. Kloetzel K, da Silva JR. Schistosomiasis mansoni acquired in adulthood: behavior of egg counts and the intradermal test. Am J Trop Med Hyg. 1967;16:167-9.

8. Clarke VD. The influence of acquired resistance in the epidemiology of bilharziasis. Cent Afr J Med. 1966;12:1-30.

9. De V. Clarke V. Evidence of the development in man of acquired resistance to infection of *Schistosoma* spp. The Central African Journal of Medicine. 1966;12:1-3.

10. Fisher MB. A study of the schistosomiasis of the Stanleyville district of the Belgian Congo. Transactions of the Royal Society of Tropical Medicine and Hygiene. 1934;28:277-306.

11. Li YS, Sleigh AC, Ross AG, Williams GM, Tanner M, McManus DP. Epidemiology of Schistosoma japonicum in China: morbidity and strategies for control in the Dongting Lake region. Int J Parasitol. 2000;30:273-81.

12. Mutapi F, Ndhlovu PD, Hagan P, Woolhouse ME. A comparison of humoral responses to Schistosoma haematobium in areas with low and high levels of infection. Parasite Immunol. 1997;19:255-63.

13. Sturrock RF, Kimani R, Cottrell B, Butterworth A, Seitz HM, Sinongok TK, et al. Observations on possible immunity to reinfection among Kenyan schoolchildren after treatment for *Schistosoma mansoni*. Transactions of the Royal Society of Tropical Medicine and Hygiene. 1983;77:363-71.

14. Horowitz S, Tarrab-Hazdai R, Eshhar Z, Arnon R. Anti-schistosome monoclonal antibodies of different isotypes--correlation with cytotoxicity. EMBO J. 1983;2:193-8.

15. Fulford AJ, Butterworth AE, Sturrock RF, Ouma JH. On the use of age-intensity data to detect immunity to parasitic infections, with special reference to Schistosoma mansoni in Kenya. Parasitology. 1992;105 ( Pt 2):219-27.

16. Mitchell KM, Mutapi F, Mduluza T, Midzi N, Savill NJ, Woolhouse ME. Predicted impact of mass drug administration on the development of protective immunity against Schistosoma haematobium. PLoS Negl Trop Dis. 2014;8:e3059.

17. Butterworth AE, Capron M, Cordingley JS, Dalton PR, Dunne DW, Kariuki HC, et al. Immunity after treatment of human schistosomiasis mansoni. II. Identification of resistant individuals, and analysis of their immune responses. Trans R Soc Trop Med Hyg. 1985;79:393-408.

18. Jiz M, Friedman JF, Leenstra T, Jarilla B, Pablo A, Langdon G, et al. Immunoglobulin E (IgE) responses to paramyosin predict resistance to reinfection with Schistosoma japonicum and are attenuated by IgG4. Infect Immun. 2009;77:2051-8.

19. Wilson S, Jones FM, Kenty LC, Mwatha JK, Kimani G, Kariuki HC, et al. Posttreatment changes in cytokines induced by Schistosoma mansoni egg and worm antigens: dissociation of immunity- and morbidity-associated type 2 responses. J Infect Dis. 2014;209:1792-800.

20. Mutapi F, Ndhlovu PD, Hagan P, Woolhouse ME. A comparison of re-infection rates with Schistosoma haematobium following chemotherapy in areas with high and low levels of infection. Parasite Immunol. 1999;21:253-9.

21. van den Biggelaar AH, Borrmann S, Kremsner P, Yazdanbakhsh M. Immune responses induced by repeated treatment do not result in protective immunity to Schistosoma haematobium: interleukin (IL)-5 and IL-10 responses. J Infect Dis. 2002;186:1474-82.

22. Smithers SR, Terry RJ. Resistance to experimental infection with Schistosoma mansoni in rhesus monkeys induced by the transfer of adult worms. Trans R Soc Trop Med Hyg. 1967;61:517-33.

23. Mbanefo EC, Huy NT, Wadagni AA, Eneanya CI, Nwaorgu O, Hirayama K. Host determinants of reinfection with schistosomes in humans: a systematic review and meta-analysis. PLoS Negl Trop Dis. 2014;8:e3164.

24. Mitchell KM, Mutapi F, Woolhouse ME. The predicted impact of immunosuppression upon population age-intensity profiles for schistosomiasis. Parasite Immunol. 2008;30:462-70.

25. Mitchell KM, Mutapi F, Savill NJ, Woolhouse ME. Protective immunity to Schistosoma haematobium infection is primarily an anti-fecundity response stimulated by the death of adult worms. Proc Natl Acad Sci U S A. 2012;109:13347-52.

26. Bushara HO, Majid AA, Saad AM, Hussein MF, Taylor MG, Dargie JD, et al. Observations on cattle schistosomiasis in the Sudan, a study in comparative medicine. II. Experimental demonstration of naturally acquired resistance to Schistosoma bovis. Am J Trop Med Hyg. 1980;29:442-51.

27. Polman K, Stelma FF, Le Cessie S, De Vlas SJ, Falcao Ferreira ST, Talla I, et al. Evaluation of the patterns of Schistosoma mansoni infection and re-infection in Senegal, from faecal egg counts and serum concentrations of circulating anodic antigen. Ann Trop Med Parasitol. 2002;96:679-89.

28. Xu CB, Verwaerde C, Grzych JM, Fontaine J, Capron A. A monoclonal antibody blocking the Schistosoma mansoni 28-kDa glutathione S-transferase activity reduces female worm fecundity and egg viability. Eur J Immunol. 1991;21:1801-7.

29. Cheever AW, Duvall RH. Single and repeated infections of grivet monkeys with Schistosoma mansoni: parasitological and pathological observations over a 31-month period. Am J Trop Med Hyg. 1974;23:884-94.

30. Coyne MJ, Smith G. The regulation of mortality and fecundity in Schistosoma mattheei following a single experimental infection in sheep. Int J Parasitol. 1991;21:877-82.

31. Cheever AW, Kamel IA, Elwi AM, Mosimann JE, Danner R. Schistosoma mansoni and S. haematobium infections in Egypt. II. Quantitative parasitological findings at necropsy. Am J Trop Med Hyg. 1977;26:702-16.

32. Agnew AM, Murare HM, Doenhoff MJ. Immune attrition of adult schistosomes. Parasite Immunol. 1993;15:261-71.

33. Kurtis JD, Friedman JF, Leenstra T, Langdon GC, Wu HW, Manalo DL, et al. Pubertal development predicts resistance to infection and reinfection with Schistosoma japonicum. Clin Infect Dis. 2006;42:1692-8.

34. Fitzsimmons CM, Jones FM, Pinot de Moira A, Protasio AV, Khalife J, Dickinson HA, et al. Progressive cross-reactivity in IgE responses: an explanation for the slow development of human immunity to schistosomiasis? Infect Immun. 2012;80:4264-70.

35. Dunne DW, Bickle QD, Butterworth AE, Richardson BA. The blocking of human antibody-dependent, eosinophil-mediated killing of Schistosoma mansoni schistosomula by monoclonal antibodies which cross-react with a polysaccharide-containing egg antigen. Parasitology. 1987;94 ( Pt 2):269-80.

36. Holford TR, Hardy RJ. A stochastic model for the analysis of age-specific prevalence curves in schistosomiasis. J Chronic Dis. 1976;29:445-58.

37. Chan MS, Guyatt HL, Bundy DA, Booth M, Fulford AJ, Medley GF. The development of an age structured model for schistosomiasis transmission dynamics and control and its validation for Schistosoma mansoni. Epidemiol Infect. 1995;115:325-44.

38. Chan MS, Mutapi F, Woolhouse ME, Isham VS. Stochastic simulation and the detection of immunity to schistosome infections. Parasitology. 2000;120 ( Pt 2):161-9.

39. Bailey RC, Homer LD. Interpretation of a stochastic model for analysis of age-specific prevalence curves in schistosomiasis. J Chronic Dis. 1978;31:455-9.

40. Cheever AW. A quantitative post-mortem study of Schistosomiasis mansoni in man. Am J Trop Med Hyg. 1968;17:38-64.

41. Brown SP, Grenfell BT. An unlikely partnership: parasites, concomitant immunity and host defence. Proc Biol Sci. 2001;268:2543-9.

42. Ashby B, King KC. Friendly foes: The evolution of host protection by a parasite. Evol Lett. 2017;1:211-21.

43. Wilson S, Jones FM, van Dam GJ, Corstjens PL, Riveau G, Fitzsimmons CM, et al. Human Schistosoma haematobium antifecundity immunity is dependent on transmission intensity and associated with immunoglobulin G1 to worm-derived antigens. J Infect Dis. 2014;210:2009-16.

44. Polman K, Stelma FF, Gryseels B, Van Dam GJ, Talla I, Niang M, et al. Epidemiologic application of circulating antigen detection in a recent Schistosoma mansoni focus in northern Senegal. Am J Trop Med Hyg. 1995;53:152-7.

45. Smithers SR. Acquired resistance to Schistosoma mansoni in the rhesus monkey (Macaca mulatta). Ann Soc Belges Med Trop Parasitol Mycol. 1967;47:87-95.

46. Smithers SR, Terry RJ, Hockley DJ. Host antigens in schistosomiasis. Proc R Soc Lond B Biol Sci. 1969;171:483-94.

47. Damian RT, Rawlings CA, Bosshardt SC. The fecundity of Schistosoma mansoni in chronic nonhuman primate infections and after transplantation into naive hosts. J Parasitol. 1986;72:741-7.

48. El Ridi R, Tallima H, Selim S, Donnelly S, Cotton S, Gonzales Santana B, et al. Cysteine peptidases as schistosomiasis vaccines with inbuilt adjuvanticity. PLoS One. 2014;9:e85401.

49. Hassan SI, Botros SS, el-Nahal HM, Azab ME, Shaker ZA, el-Garem AA. Effect of specific chemotherapy on the immune response and resistance to reinfection in experimental Schistosomiasis mansoni. Int J Immunopharmacol. 1990;12:207-15.

50. Ribeiro F, Mello RT, Tavares CA, Kusel JR, Coelho PM. Synergistic action of praziquantel and host specific immune response against Schistosoma mansoni at different phases of infection. Rev Inst Med Trop Sao Paulo. 2004;46:231-3.

51. Salim A, Al-Humiany A. Concomitant immunity to *Schistosoma mansoni* in mice. Turkiye Parazitol Derg. 2013;37:19-22.

52. Webbe G, James C. Letter: Acquired resistance to Schistosoma haematobium in the baboon (Papio anubis). Trans R Soc Trop Med Hyg. 1973;67:151-2.

53. Agnew A, Fulford AJ, Mwanje MT, Gachuhi K, Gutsmann V, Krijger FW, et al. Age-dependent reduction of schistosome fecundity in Schistosoma haematobium but not Schistosoma mansoni infections in humans. Am J Trop Med Hyg. 1996;55:338-43.

54. Chandiwana SK, Woolhouse ME, Bradley M. Factors affecting the intensity of reinfection with Schistosoma haematobium following treatment with praziquantel. Parasitology. 1991;102 Pt 1:73-83.
